# Photoluminescence and electronic transitions in cubic silicon nitride

**DOI:** 10.1038/srep18523

**Published:** 2016-01-04

**Authors:** Luc Museur, Andreas Zerr, Andrei Kanaev

**Affiliations:** 1 Laboratoire de Physique des Lasers, CNRS, Université Paris 13, Sorbonne Paris Cité, 93430 Villetaneuse, France; 2 Laboratoire des Sciences des Procédés et des Matériaux, CNRS, Sorbonne Paris Cité, Université Paris 13, 93430 Villetaneuse, France

## Abstract

A spectroscopic study of cubic silicon nitride (γ-Si_3_N_4_) at cryogenic temperatures of 8 K in the near IR - VUV range of spectra with synchrotron radiation excitation provided the first experimental evidence of direct electronic transitions in this material. The observed photoluminescence (PL) bands were assigned to excitons and excited 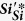 and 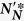 centers formed after the electron capture by neutral structural defects. The excitons are weakly quenched on neutral 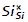 and strongly on charged 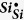 defects. The fundamental band-gap energy of 5.05 ± 0.05 eV and strong free exciton binding energy ~0.65 eV were determined. The latter value suggests a high efficiency of the electric power transformation in light in defect-free crystals. Combined with a very high hardness and exceptional thermal stability in air, our results indicate that γ-Si_3_N_4_ has a potential for fabrication of robust and efficient photonic emitters.

After heating/cooling and transportation, lighting appears to be the most energy consuming sector of the mankind activity where a considerable progress was recently achieved by development of light emitting diodes (LEDs)^1^. The main part of the industrially used materials for fabrication of LEDs is based today on binary or ternary compounds of the group 13 and 15 elements such as GaN, InN, AlN, GaAs, GaP etc.^1^,^2^. These compounds have a number of disadvantages: All of them are relatively expensive since the group 13 elements are rare in the nature, some of them are considered as toxic (e.g. GaAs), and almost all of them have relatively low efficiencies due to a low exciton binding energy. Moreover, they are not stable against hydrolysis and oxidation in air, especially at elevated temperatures. Few other emitting materials less sensitive to the heat release (ZnO and hBN) are under studies^3^,^4^.

An interesting alternative to the contemporary materials are novel high-pressure (HP) nitrides of the group 14 elements having cubic spinel structure, γ-M_3_N_4_ where M = Si, Ge, or Sn, and their solid solutions^5^,^6^. γ-Si_3_N_4_ obtained first via a chemical reaction of the elements at high pressures and temperatures^7^ is the most prominent member of the family, which synthesis was performed applying different static and dynamic HP techniques and chemical reaction paths^5^,^8^. γ-Si_3_N_4_ was predicted to have a large direct band gap, its hardness of HV = 30–43 GPa^9^,^10^ is surpassed only by diamond and cubic BN, and it has an exceptional thermal stability in air up to 1400 °C^10^, which significantly exceeds those of any known LED material. The thermal stability of the γ-phase is similar to that of the low pressure α- and β-phases where a thin O-rich passivating layer preserves further diffusion of oxygen into the material bulk. The exceptional properties combination suggests suitability of this spinel nitride for a variety of optoelectronic application in demanding environment conditions with the emphasis on high power-density lighting.

Despite of much effort in the material synthesis and understanding of structural and mechanical properties, electronic structure and optical properties of γ-Si_3_N_4_, which are of key importance for the lighting applications, have not been yet adequately addressed. Only a few related studies have been performed until now. The first theoretical calculations of the structure and properties of cubic spinel nitrides using local density approximation (LDA) have predicted γ-Si_3_N_4_ to be a direct band-gap semiconductor with the interband energy of E_g_ = 3.45 eV^11^,^12^,^13^. Ding *et al.*^14^ have reported the theoretical band-gap energy of γ-Si_3_N_4_ to be 3.58 and 3.40 eV using respectively GGA and LDA methods. However, DFT-LDA calculations were found to underestimate considerably the band-gap energy and the very recent G_0_W_0_ calculations performed by Chu *et al.*^15^ have resulted in a higher value of E_g_ = 4.87–4.89 eV. In the same time, it was underlined that the obtained value is very sensitive to the lattice parameter, which relative variation e.g. by 3% shifts this energy by 0.64 eV. Very recently, Caskey *et al.*^16^ have reported the indirect transitions slightly lower ~0.07 eV (2.8 kT at 300 K) than the direct transitions (5.16 eV) in this material. An important drawback when compared with uncertainties in the band gap size is the absence of theoretical and experimental information, which would allow concluding about the efficiency of energy conversion in a LED based on this material. One of the key values is binding energy of free exciton, which is related to the probability of radiative relaxation of an electron from the conduction to valence band. This physical parameter can be calculated from first principles for simple structures^17^,^18^ with a few atoms in the unit cell but not yet for a compound with a cubic spinel structure where the unit cell contains 56 atoms. A more simplified calculation assuming hydrogenic-type exciton quasiparticle gave the exciton binding energy of 333 meV^6^, which is one order of magnitude higher than that in GaN (26 meV^19^). However, the hydrogenic-type model adapted for Wannier excitons with a relatively small binding energy is not adequate to γ-Si_3_N_4_.

The experimental measurements of the band gap energy were previously undertaken using soft-x-ray absorption and emission spectroscopy to probe the localized partial density of occupied and unoccupied states near the Fermi level and have resulted in E_g_ = 4.30 ± 0.25 eV^20^. More recently using the same method, Boyko *et al.*^6^,^21^ have reported the band gap energy of 4.7 eV, excluding the core hole shift between the measured band edges, and E_g_ = 4.8 ± 0.2 eV after the correction based on the ground state and excited state DFT calculations. These remain today the only two reported experimental values in literature. The information about type of the electronic transitions, exciton binding energy and energy transfer processes in the material, which are of key importance for understanding of its suitability for photonic applications, cannot be accessed by this method. No direct measurements not requiring theoretical correction and/or intuitive extrapolations of the experimental points have been reported until now.

In this communication we report on the first investigation of photoluminescence (PL) properties and optical electronic transitions in γ-Si_3_N_4_ by time- and energy resolved spectroscopy method. Based on the experimental results, we discuss the direct gap electronic transitions and exciton binding energy, which is large when compared with any other LED material having a direct band gap in the blue-UV wavelength region.

## Methods

The experiments were performed at the SUPERLUMI station^22^ of HASYLAB at synchrotron DESY. Briefly, samples were cooled down to 8 K and irradiated by monochromatized (Δλ = 0.3 nm) synchrotron radiation (SR) under high vacuum (~10^−9^ mbar). The PL spectra were measured in the UV-visible-nearIR and VUV spectral range using ARC monochromator with a CCD analyzer sensitive in the spectral range of 200–1000 nm. The photoluminescence excitation (PLE) spectra were measured in the UV-VUV spectral range in short Δt_1_ = 3–8 ns and long Δt_2_ = 50–100 ns time windows with respect to the excitation SR pulse of 130 ps duration. The recorded spectra were corrected for the SR intensity and primary monochromator transmission. The PL decay curves were measured with a photomultiplier R6358 (Hamamatsu) sensitive in the spectral range 185–900 nm.

The material, γ-polymorph of Si_3_N_4_, was synthesized in a high-pressure multianvil apparatus according to a method described in ref. 23. Its crystalline structure and composition were confirmed by X-ray diffraction and Raman spectroscopy. Using this starting material, two samples were prepared: the first one was of cylindrical shape of 1 mm diameter and similar thickness and the second one was compacted in a hole of 150 μm in diameter drilled in a metallic foil of 50 μm thickness. Additionally, the second sample was exposed to an electron beam of 20 keV and 7–9 nA for 7 min in a SEM prior to the SR measurements carried out within of one week after the exposure. This e-beam irradiation produced neither visible coloration nor modification of the sample crystallinity. During the spectroscopic measurements at HASYLAB both samples were directly mounted on the cryostat and their temperature continuously monitored in the range between 8 and 300 K.

## Results

**Figure 1:**
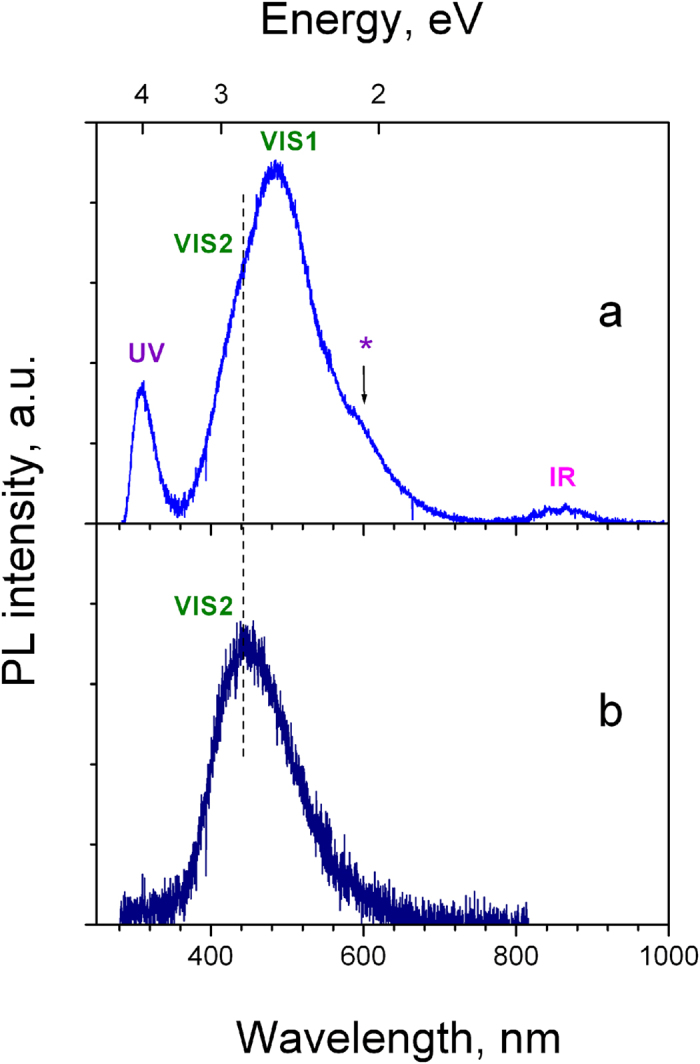
PL spectra of freshly prepared (**a**) and e-beam irradiated (**b**) γ-Si_3_N_4_ samples excited with SR-photons at 237 nm (T = 8K). The sign (*) indicates 2^nd^ order of the UV band.

**Figure 2:**
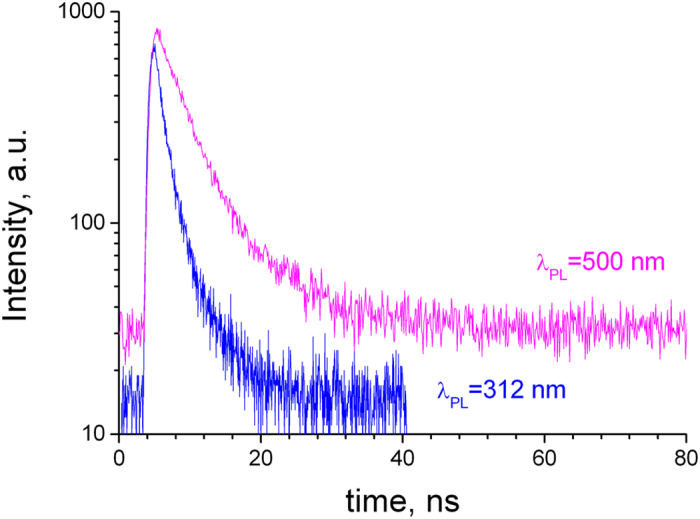
PL decay of γ-Si_3_N_4_ (λ_exc_ = 237 nm, T = 8K).

PL and PLE spectra of the untreated and e-beam irradiated γ-Si_3_N_4_ samples are shown in Fig. 1. Four bands in the ultraviolet (UV), visible (VIS1 and VIS2) and near infrared (IR) spectral regions were observed, as indicated in the figure. The UV and VIS1 emissions are short-lived (sub- and nanosecond range), while VIS2 and IR are long-lived (longer than one microsecond). The PL decay curves of the UV (λ_PL_ = 312 nm) and VIS1 (λ_PL_ = 500 nm) bands at T = 8 K are shown in Fig. 2. Both PL decays are mainly monoexponential and, consequently, can be characterized by time constants of respectively 1.4 and 4.4 ns (confidence range of these measurements is about 50 ps). We notice that the own photomultiplier response time is ~1.5 ns. Therefore, one can conclude about a very short picosecond lifetime of the excited-state responsible for the UV emission (τ ≤ 100 ps).

**Figure 3:**
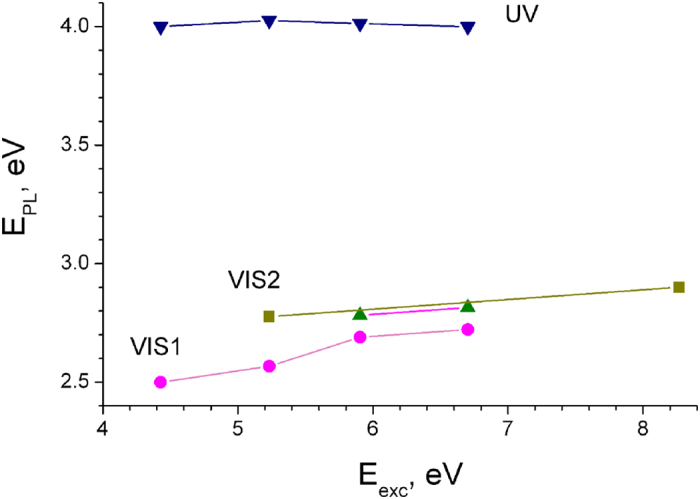
Spectral maxima of principal luminescence bands of γ-Si_3_N_4_ on excitation energy (T = 8K): UV (▾), VIS1 (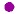) and VIS2 (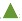, 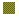) in fresh (▾, 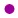, 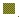) and e-beam irradiated (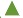) samples. The lines connecting the experimental points serve as guides for the eyes.

The spectral position of VIS1 and VIS2 PL bands shifts when the excitation energy changes from 4.4 eV to 8.4 eV, as shown in Fig. 3. Such band shifting is characteristic of the Raman regime of radiative transitions involving charged donor-acceptor pair (DAP) levels and is explained by a neutralization of charged defects^24^. However, the slope of E_PL_ vs E_exc_ dependence is more than one order of magnitude smaller compared to that previously observed for shallow DAPs^24^. Moreover, homogeneous spectral nature of VIS1 band and no transition from Raman to the photoluminescence regime make the above supposition doubtful.

In contrast to the visible PL, the spectral position of UV band (4.0 eV) remains stable when the excitation energy changes. This fact associated with a very short lifetime, constancy of line-shape with temperature and specific PLE spectra with the large Stokes energy shift of PL band (will be discussed below) allow us to assign this PL band to self-trapped exciton.

It appears that VIS1 and VIS2 bands have different origins. This conclusion is supported by their different lifetimes and different variation of spectral position and intensity when the excitation photon energy increases. In fact, VIS1 band has the nanosecond lifetime (see Fig. 2) and is more intense at long-wave excitation, while VIS2 band has a long (microsecond or longer) lifetime and shows up and dominates the spectra at excitations above 5 eV. Moreover according to Fig. 1b, both UV and VIS1 bands disappear after the e-beam irradiation, while VIS2 band remains. The last emission may therefore belong to a charged state, while charged states or structural defects induced by e-beam may quench the UV and VIS1 emissions.

**Figure 4:**
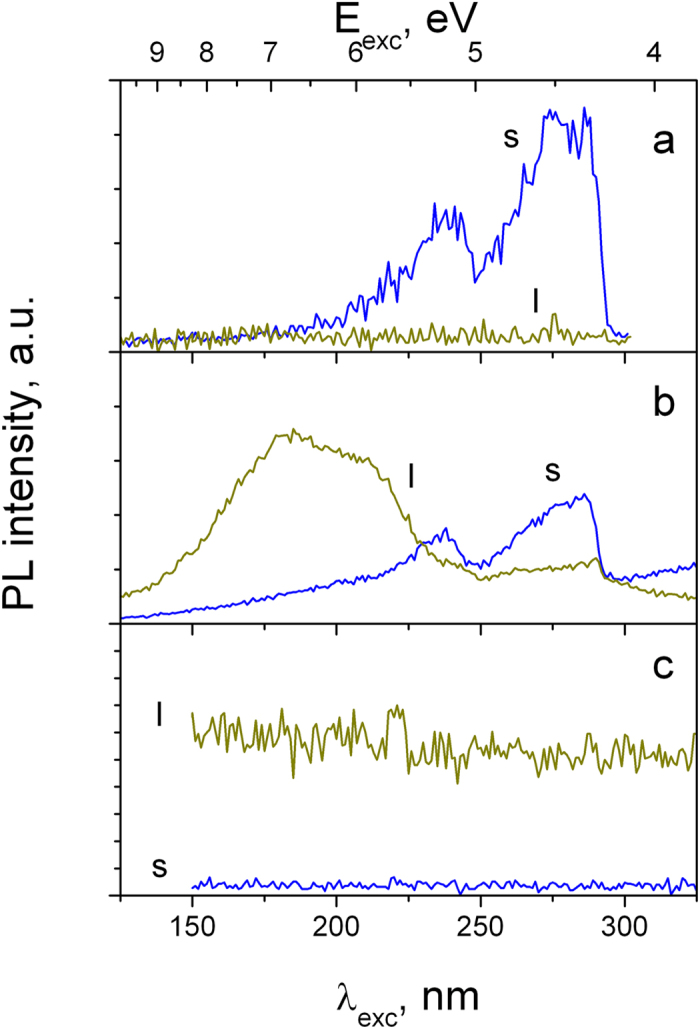
PLE spectra of short (s) and long (l) PL decay of γ-Si_3_N_4_ at 307 (a), 450 (b) and 850 (c) nm (T = 8K).

Further support to the above supposition about different natures of VIS1 and VIS2 bands follows from their excitation spectra presented in Fig. 4, where a perfect correlation between PLE of UV and VIS1 bands can be recognized, which contrasts to VIS2. We therefore assume a common origin of UV and VIS1 bands and assign the energy lower VIS1 band to the trapped exciton. The large Stokes shift (0.4 eV) of UV PL band and its relatively large bandwidth (full width half maximum 0.4 eV) allows its assignment to the self-trapped exciton formed as a result of a strong exciton-phonon coupling in the material. A similar feature has been recently observed in hBN crystals^25^,^26^. The assignment of VIS2 band can be done based on literature data reported on the amorphous silicon nitride (a-Si_3_N_4_). Indeed, the green PL of a-Si_3_N_4_ is known^27^,^28^ and has been assigned to phosphorescence of excited metastable paramagnetic nitrogen anion radical 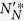 as denoted in Kröger-Vink notation. This emission is excited in our samples with photons having energy above 5 eV (*l* component in Fig. 4b). The weak seeming correlation between PLE spectra of the anion (*l*) and trapped exciton (*s*) components in Fig. 4b is explained by their partially overlapped PL bands (VIS1 and VIS2).

**Figure 5:**
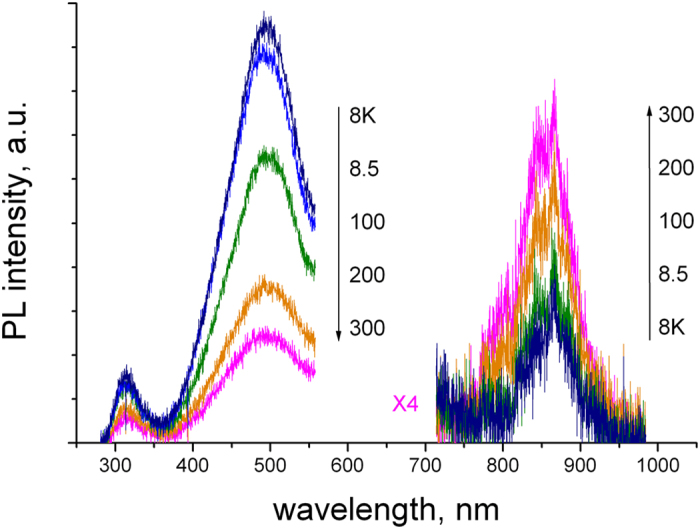
PL spectra of γ-Si_3_N_4_ for temperatures 8.2, 8.5, 100, 200 and 300 K (λ_exc_ = 237 nm).

Understanding of the origin of the IR emission is not straightforward. Because of the low intensity, its PLE spectrum is noisy that does not permit the comparison with the ultraviolet and visible bands. It looks featureless and is characterized by a long PL decay (Fig. 4c). However, a comparison is possible based on the thermal behavior of these emissions as shown in Fig. 5. Indeed, the intensities of both UV and VIS1 bands decrease with an increase of temperature, contrary to that of the IR emission which increases. This correlation suggests a common energy for the PL activation process of ΔE≈30 meV, which was determined by us assuming the UV intensity decrease due to dissociative decay of the exciton (supplementary information, S1). According to our assignment, the interaction between the excited states results in the energy transfer from the free exciton (denoted as X^*^) to IR emitting state, which is obviously accompanied by the UV (self-trapped exciton) and VIS1 (bound exciton) intensities decrease. Because of absence of any correlations between IR and VIS2 emissions, we disregard nitrogen radical as a possible origin of the IR emission and tentatively assign it to excited silicon radical 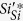.

**Figure 6:**
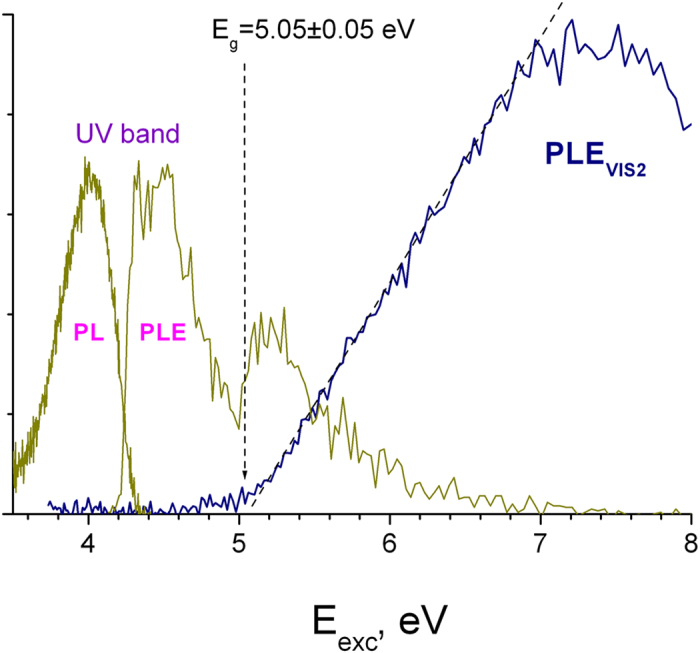
PLE spectrum of dominant long 450 nm PL decay (PLE_VIS2_) of e-beam irradiated γ-Si_3_N_4_ (T = 8K). PL and PLE spectra of short-lived UV band in non-irradiated γ-Si_3_N_4_ are also included.

A comparison between the PL and PLE spectra of UV and VIS2 bands permits determination of the band-gap energy (E_g_) of γ-Si_3_N_4_, as shown in Fig. 6. The PLE spectrum of UV band has two characteristic maxima related to the direct free exciton excitation (4.4 eV) and exciton formation due to the recombination of low kinetic energy electron-hole pairs at the conduction band (CB) bottom: E_exc_≈5.2 eV. Similar spectral features have been previously observed, e.g. in α-Al_2_O_3_^30^. Accordingly when the photon energy further increases above E_g_, the excitation efficiency decreases due to an increased kinetic energy of the photoinduced charges, which makes their separation more probable and prevents exciton formation. In framework of this model, the band-gap energy can be assigned to the minimum of the exciton PLE situated between the two spectral maxima at 4.4 eV and 5.2 eV. Another possibility is to use the PLE onset of VIS2 band of the 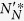 centers, which are populated after the CB electrons relaxation, as show Fig. 4b and, more clearly, Fig. 6. The better precision obtained with the e-beam irradiated sample is due to the fact that here, in contrast to the original material, VIS2 emission dominates PL spectra (Fig. 1). The both approaches converge to the same band gap energy of E_g_ = 5.05 ± 0.05 eV. This value is larger compared to those previously extracted from soft-x-ray absorption and emission spectroscopy^6^,^20^,^21^. However taking into account the confidence range, the result reported by Boyko *et al.*^6^ spans to the limit of our directly obtained E_g_. In view of the band gap data discrepancy reported in previous theoretical studies^11^,^12^,^13^,^14^,^15^,^16^, the reported direct transition energy may be useful for the calculations adjustment.

The relative position of the free exciton PLE maximum at 4.35 eV and E_g_ allows to conclude about the large binding energy D_e_≈0.65 eV of free exciton in γ-Si_3_N_4_, which is characteristic of Frenkel type. The apparent exciton deactivation temperature ~350 K (30 meV) obtained from Fig. 5 cannot be explained by D_e_ and reflects the energy transfer to a quasi-resonant 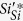 defect level emitting in the IR range of spectrum.

**Figure 7:**
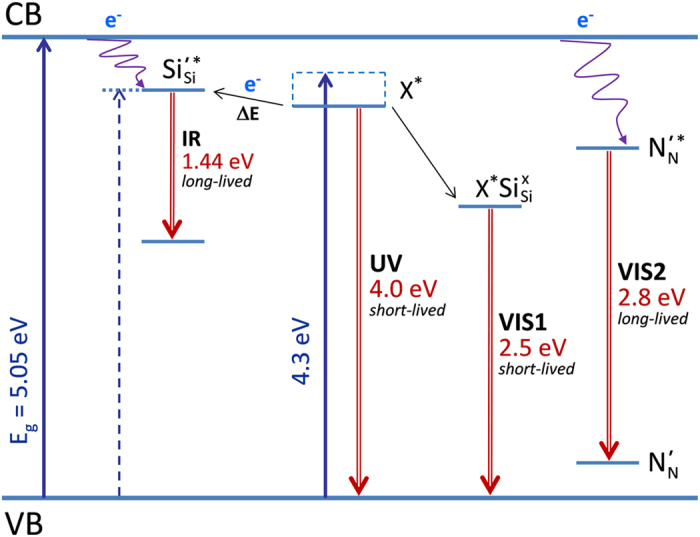
Schema of the radiative processes in γ-Si_3_N_4_.

The schema of radiative processes in γ-Si_3_N_4_ with photon excitation is shown in Fig. 7. The bonding of Si_3_N_4_ can be described in terms of the hybrid interaction of sp^3^ on silicon atoms with sp^2^ on adjacent nitrogen atoms. The conduction band (CB) is Si *s* like and valence band (VB) is formed of N *pπ* states^31^,^32^. We assume that neutral defects 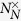 and 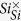 dominate in the untreated Si_3_N_4_ sample. In contrast, charged defects participate in energy and charge relaxation in samples exposed to the e-beam irradiation. The global crystal electro-neutrality suggests the formation of compensated centers, which are supposed to be 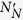 and 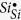. Indeed since ground-state paramagnetic anions 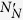 are necessary produced after the CB electrons relaxation, they can be compensated by 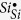 cations. Both 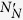 and 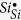 centers are metastable and survive in *a*-Si_3_N_4_ samples during months^29^.

The reactions involving relevant exciton, neutral and charged states are:


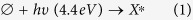



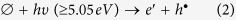



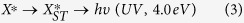



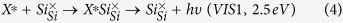



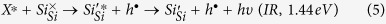



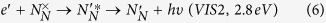


PL of free excitons is not observed since they undergo an efficient self-trapping (3) and binding to neutral silicon defects (4). After e-beam irradiation, a strong concentration of charged defects appears and the relaxation channels change. Those related to excitons disappear probably due to the quenching on silicon cations, which recover electro-neutrality


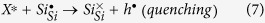



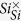
 do not emit photons and induce PL after the binding of free excitons X^*^ according to (4). The only significant remaining PL band in these conditions is VIS2, which originates from the CB electrons transfer to neutral nitrogen centers (6). The analysis of the VIS2 band intensity in non-irradiated and e-beam irradiated γ-Si_3_N_4_ suggests a partial conversion of 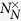 to ground-state 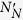 in course of the e-beam irradiation and radiative relaxation according to process (6). At the same time, compensated 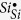 centers appear that quench the exciton-related emissions.

We notice that our assignment of the IR emitting state needs a confirmation. In fact, Belyi *et al.*^29^ have previously suggested shallow electron traps close to CB (4.4–4.7 eV) to be related to nitrogen cation radicals, which recombine to form the ground-state anion radical accompanied by the emission of 3.8 eV photons. Because of the observed short exciton lifetime, characteristic spectral features and energy transfer to the IR emitting state, we cannot adopt this hypothesis and assign the relevant defect level to Si^×^_Si_ situated close to CB. The band-gap narrowing has been previously attributed in non-stoichiometric SiN_x_ samples to a decrease in the Si-Si coordination number^31^. Discrete silicon states may therefore appear close to CB of γ-Si_3_N_4_. The IR emission in γ-Si_3_N_4_ may also be explained by rare inclusions of silicon nanoclusters of size ~3–4 nm, which provide intense PL in the vicinity of 850 nm^33^.

### Perspectives

The obtained spectroscopic results indicate that the major channel of the non-radiative energy losses is the exciton quenching on structural defects, probably related to broken and/or non-stoichiometric silicon bonds. These losses may be overcome by improvement of the γ-Si_3_N_4_ crystals quality and optimizing the synthesis process. On the other hand, the unusually short picosecond radiative lifetime of exciton (also observed in hexagonal boron nitride, hBN^25^,^26^,^34^) suggests less strict requirements to the defects number density in the crystal, which facilitates the fabrication process design. Moreover, the strong exciton binding energy 0.65 eV suggests the exciton stability in almost whole range of the material thermal stability up to 1400 °C^10^. We notice that the obtained here exciton binding energy of γ-Si_3_N_4_ is almost 2 times higher than that earlier reported in ref. 6, which makes appealing new theoretical studies in the field.

γ-Si_3_N_4_ exhibits a much stronger exciton binding energy when compared to GaN (26 meV)^19^, ZnO (60 meV)^3^ and similar to that of hBN (0.7 eV)^34^,^35^,^36^. One can estimate the exciton emission efficiency factor 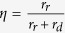, where 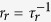 and 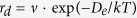 are respectively radiative and dissociation rates of exciton. The radiative exciton lifetime τ_r_ ~ 100 ps was obtained in this work. The frequency ν = 6∙10^13^ s^−1^ can be estimated in the framework of the Onsager-Braun model (supplementary information, S2). As a result, η attains almost 100% in γ-Si_3_N_4_, while it is ~0.01% in GaN (free exciton lifetime in GaN is ~295 ps^37^).

Recently, Watanabe *et al.*^38^ have reported that a mere pressure applied between two fingers can easily deform hBN crystal, which results in a dramatic degradation of the band-edge luminescence due to the exciton decay on lattice dislocations^39^. An ordinary mechanical constraint inevitably induces such effect that prevents obtaining an efficient emission. In contrast, the exciton emission in hard γ-Si_3_N_4_ crystals is expected to be much less sensitive to mechanical constraints and more stable compared to hBN. A high exciton emission efficiency can therefore be expected for γ-Si_3_N_4_ with sufficiently low defect concentration. Consequently, this material has a potential for fabrication of robust and efficient photonic emitters.

The existing theoretical calculations suggest direct electronic transitions for other members of the spinel nitride family, such as γ-Ge_3_N_4_, γ-Sn_3_N_4_ and solid solutions γ-(Si_1-x_, Ge_x_)_3_N_4_ and γ-(Ge_1-x_, Sn_x_)_3_N_4_, known to have elevated hardness and thermal stability. The estimation of the band gap and exciton binding energy (in the model of the hydrogen-like quasiparticle) showed for the other members a progressive decrease from the highest value for γ-Si_3_N_4_ (E_g_ ~ 5 eV and D_e_ ~ 333 meV) to the lowest value for γ-Sn_3_N_4_ (E_g_ ~ 1.5 eV and D_e_ ~ 69 meV). In view of these and our present results, we expect for any member of the spinel nitride family its exciton binding energy to exceed significantly that of the competing materials with similar band gap energy. As a consequence, a LED based on a spinel nitride would have, for any radiation wavelength, a significantly better performance and efficiency of the electric energy conversion in light when compared the LEDs based on the presently used materials. The existence of solid solutions with a smoothly changing band gap, confirmed experimentally for γ-(Si_1-x_, Ge_x_)_3_N_4_ and expected for γ-(Ge_1-x_, Sn_x_)_3_N_4_^6^, could make possible fabrication of efficient light sources ideally imitating the solar light. The recent report on a reproducible deposition of γ-Sn_3_N_4_ via an economic deposition method involving magnetron sputtering shows a promising direction for the industrial fabrication of novel advanced light sources^16^.

The present results show the importance of further theoretical and experimental studies of the synthesis processes of γ-Si_3_N_4_ and other spinel nitrides sighting tuning of their electronic band structure and, accordingly, optoelectronic properties.

## Conclusions

In the present study, we have examined time- and energy- resolved luminescence of the spinel phase of silicon nitride (γ-Si_3_N_4_) at cryogenic temperatures in the near-IR-VUV range of spectra with SR excitation. Several photoluminescence bands were observed. The principal short-lived UV (4.0 eV) and visible VIS1 (2.5 eV) bands were assigned respectively to self-trapped and bound excitons and long-lived visible VIS2 band (2.8 eV) was assigned to paramagnetic anions 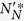. The results provide the first experimental confirmation to the theoretical prediction of the direct electronic transitions in γ-Si_3_N_4_. The fundamental band-gap energy of E_g_ = 5.05 ± 0.05 eV was obtained from the analysis of experimental PL and PLE spectra. The strong binding energy ~0.65 eV of free exciton suggests its high thermal stability and emission efficiency at temperatures up to 1000 °C provided the γ-Si_3_N_4_ crystals contain a sufficiently low concentration of defects. The obtained spectroscopic results indicate that the exciton disappears interacting with structural defects, probably related to broken and/or non-stoichiometric silicon bonds. In particular, free excitons transfer energy to neutral Si-defects (energetically situated ~30 meV higher), which emit at 1.44 eV, and they are non-radiatively quenched on silicon cations. Consequently, fabrication of the defect free crystals is a key issue in attaining efficient LEDs based on this spinel nitride.

By analogy, we expect for other members of the spinel nitride family (such as γ-Ge_3_N_4_, γ-Sn_3_N_4_ and their solid solutions γ-(Si_1-x_, Ge_x_)_3_N_4_ and γ-(Ge_1-x_, Sn_x_)_3_N_4_) direct electronic transitions tunable in energy with the composition.

## Additional Information

**How to cite this article**: Museur, L. *et al.* Photoluminescence and electronic transitions in cubic silicon nitride. *Sci. Rep.* **6**, 18523; doi: 10.1038/srep18523 (2016).

## Supplementary Material

Supplementary Information
