# Supplementary material for: Photoluminescence and electronic transitions in cubic silicon nitride
Source: Sci Rep. 2016 Jan 4;6:18523. doi: 10.1038/srep18523 (PMC4698759; doi:10.1038/srep18523)
Supplement: Supplementary Information [file srep18523-s1.pdf]

Photoluminescence and electronic transitions in cubic silicon nitride

Luc Museur[1], Andreas Zerr[2] and Andrei Kanaev[2*]

Supplementary information

S1. Activation energy of free exciton quenching

The variation of PL intensity of exciton and $Si_{Si}^{'*}$ defect in γ-Si_3N_4 versus temperature is shown in Figure S1-1. These data were corrected for the spectral sensitivity of ARC monochromator with CCD camera analyzer and the integral PL intensities were accordingly renormalized.

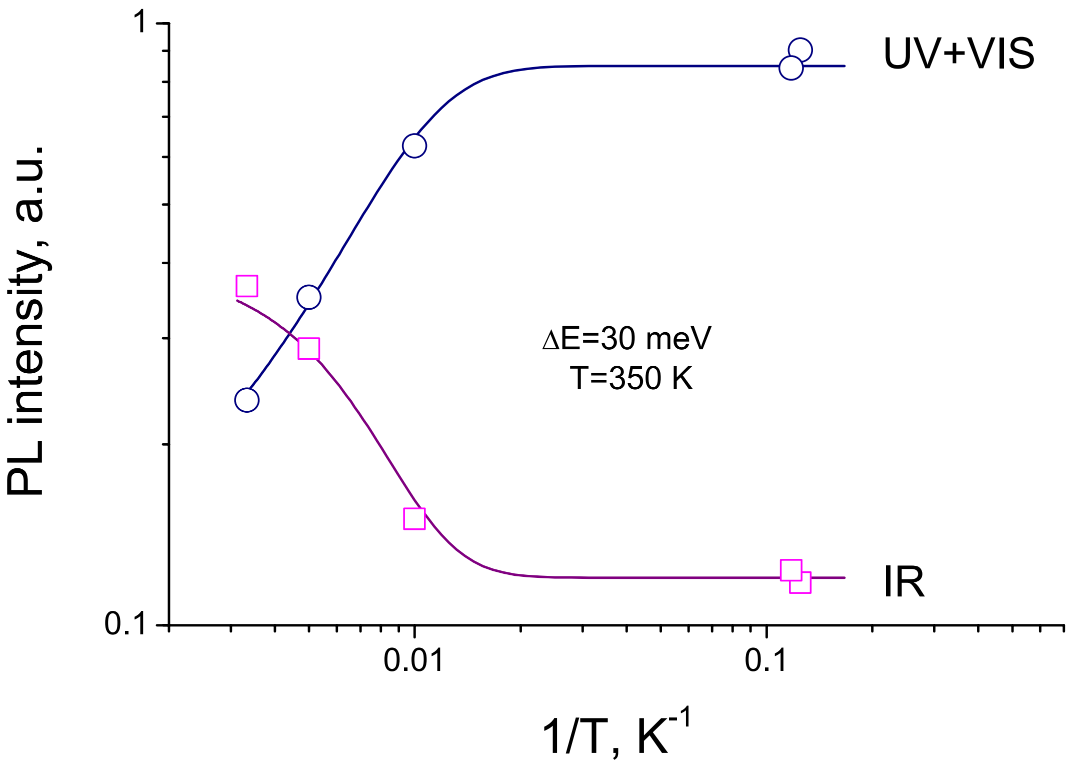

Figure S1-1: PL intensities of excitons (UV+VIS1) and $Si_{Si}^{'*}$ defects (IR) versus temperature in γ-Si_3N_4 (λ_{exc}=237 nm).

The experimental data can be described in framework of a simple model taking into account the rates of energy transfer and radiative transitions of free, self-trapped and bound excitons and defects. We assume a negligible PL of free excitons, which mainly decay through the energy transfer to the self-trapped (r_{st}) and bound (r_b) excitons and defects (r_d), which decay radiatively. Solving the differential equations kinetic describing the excited states populations, we can obtain the PL intensities of free ($I_{ex\text{-}f}$), self-trapped ($I_{ex\text{-}st}$) and bound ($I_{ex\text{-}b}$) excitons and defects (I_d):

$$I_{ex-f} = A \cdot r_r /(r_r + r_{st} + r_b + r_d) \quad \text{(S1-1)}$$

$$I_{ex-st} = A \cdot r_{st} /(r_r + r_{st} + r_b + r_d) \quad \text{(S1-2)}$$

$$I_{ex-b} = A \cdot r_b /(r_r + r_{st} + r_b + r_d) \quad \text{(S1-3)}$$

$$I_d = \gamma A \cdot r_d /(r_r + r_{st} + r_b + r_d) + B \quad \text{(S1-4)}$$

where A and B are constants that depend on the initial direct excited level populations and γ is the quantum yield of defect PL. We assign $r_d = \omega \cdot \exp(\Delta E / k_B T)$, where ω, ΔE and k_B are respectively frequency constant, activation energy and Boltzmann constant. We remark that $r_d \approx 0$ at T=8K and the free exciton lifetime $\tau^{-1} = r_r + r_{st} + r_b$. Then we obtain

$$I_{ex} = I_{ex-f} + I_{ex-st} + I_{ex-b} = A/(1 + r_d \tau) \quad \text{(S1-5)}$$

$$I_d = \gamma (A - I_{ex}) + B \quad \text{(S1-6)}$$

The fit of the experimental series with Equations (S1-5) and (S1-6) is shown in Figure S1-1 and provides the activation energy of the exciton quenching $\Delta E \approx 30$ meV (T=350 K) and $\gamma \sim 50\%$. The PL emissions perfectly correlate, which evidences the energy transfer from free excitons to $Si_{Si}^{'*}$ defects:

$$X^* + Si_{Si}^{\times} \rightarrow Si_{Si}^{'*} + h^{\bullet} \quad \text{(S1-7)}$$

A non-negligible population of $Si_{Si}^{'*}$ takes place after the excitation above the band gap $h\nu > E_g = 5.05$ eV (e.g. $h\nu = 5.23$ eV in Figure S1-1), which is due to the CB electrons attraction to neutral silicon defects:

$$e' + Si_{Si}^{\times} \rightarrow Si_{Si}^{'*} \quad \text{(S1-7)}$$

In a similar way, according to our observations, CB electrons can be attached to neutral nitrogen defects to form nitrogen anion radicals ${N_N'}^*$ emitting in the visible spectral range (VIS2 band, λ_{PL}=445 nm):

$$e' + N_N^{\times} \rightarrow {N_N'}^* \tag{S1-8}$$

S2. Free exciton dissociation rate

The exciton dissociation rate has been estimated from the Onsager-Braun model [S2-1]:

$$r_d = \nu \cdot \exp(-D_e / kT) \tag{S2-1}$$

with the frequency factor

$$\nu = 3e(\mu_e + \mu_h) / 4\pi\varepsilon a^3 \tag{S2-2}$$

where e, μ_e, μ_h, ε, a are respectively electron charge, electron and hole mobilities, dielectric constant and electron-hole pair distance.

We used the calculated values of ε=4.7 and effective hole and electron masses m_h>>m_e=0.51 [S2-2] to estimate the exciton Bohr radius a=a_B(Ry/$D_e m_e$)$^{1/2}$=0.35 nm (with Ry=13.6 eV, a_B=0.053 nm and our experimental value of D_e=0.65 eV). The electron mobility μ_e≈1 cm²/V/s measured for spinel tin nitride [S2-3] has been taken as representative for our material. As a result, we obtain from Equation (S2-2) the frequency factor ν=5.7·10^{13} s^{-1} for γ-Si_3N_4.

Accordingly, taking into account the dielectric constant ε=5.35, exciton radius a=3.29 nm [S2-4] and mobilities μ_e=1000 cm²/V/s and μ_h≤200 cm²/V/s [S2-5], we obtain ν=6.0·10^{13} s^{-1} for wurtzite GaN.

Equation (S2-1) has been used for the estimation of the exciton dissociation rates in γ-Si_3N_4 and GaN using their respective binding energies D_e=0.65 eV (present work) and 26 meV [S2-6]. In particular, at room temperature (kT≈25 meV) in γ-Si_3N_4 the exciton dissociation rates r_d=300 s^{-1} is negligible compared to the rate of radiative transitions $r_d << r_r = \tau_r^{-1}$~10^{10} s^{-1} observed in this work (τ_r~100 ps), while in GaN r_d=2·10^{13} s^{-1} >> r_{PL} (τ_{UV}=295 ps [S2-7]).

References

S2-1 Braun C. L. Electric field assisted dissociation of charge transfer states as a mechanism of photocarrier production. *J. Chem. Phys.* **80**, 4157-4161, (1984).

S2-2 Mo, S-D. et al. Interesting physical properties of the new spinel phase of Si_3N_4 and C_3N_4. *Phys. Rev. Lett.* **83**, 5046-5049 (1999).

S2-3 Caskey C. M. et al. Semiconducting properties of spinel tin nitride and other IV_3N_4 polymorphs. *J. Mater. Chem. C* **3**, 1389-1396 (2015).

S2-4 Hanada T. Basic Properties of ZnO, GaN, and Related Materials. *Advances in Materials Research* **12**, 1-19 (2009).

S2-5 *Properties of Advanced Semiconductor Materials: GaN, AlN, InN, BN, SiC, SiGe.* Levinshtein M. E., Rumyantsev S. L., Shur M. S. (Eds), Wiley 2001.

S2-6 Viswanath, A. K., Lee, J. I., Kim, D., Lee, C. R., Leem, J. Y. Exciton-phonon interactions, exciton binding energy, and their importance in the realization of room-temperature semiconductor lasers based on GaN. *Phys. Rev. B* **58**, 16333-16339 (1998).

S2-7 Bunea G. E., Herzog W. D., Ünlü M. S., Goldberg B. B., Molnar R. J. Time-resolved photoluminescence studies of free and donor-bound exciton in GaN grown by hydride vapor phase epitaxy. *Appl. Phys. Lett.* **75**, 838-840 (1999).
